# Exosomal miR-17-5p from adipose-derived mesenchymal stem cells inhibits abdominal aortic aneurysm by suppressing TXNIP-NLRP3 inflammasome

**DOI:** 10.1186/s13287-022-03037-1

**Published:** 2022-07-26

**Authors:** Jiateng Hu, Yihong Jiang, Xiaoyu Wu, Zhaoyu Wu, Jinbao Qin, Zhen Zhao, Bo Li, Zhijue Xu, Xinwu Lu, Xin Wang, Xiaobing Liu

**Affiliations:** 1 Department of Vascular Surgery, Shanghai Ninth People’s Hospital, Shanghai Jiao Tong University School of Medicine, Shanghai, China; 2 Vascular Centre of Shanghai Jiao Tong University, Shanghai, China

**Keywords:** TXNIP, Adipose-derived mesenchymal stem cells, Exosomes, microRNA-17-5p, Macrophages, Inflammation

## Abstract

**Background:**

Preclinical studies have suggested that adipose-derived mesenchymal stem cells (ADSCs) transplantation can suppress abdominal aortic inflammation and aneurysm expansion through paracrine factors. Yet, the mechanism of action is not fully understood. In the present study, we further examined the function and mechanism of ADSC-derived exosomes (ADSC-exos) and their microRNA-17-5p (miR-17-5p) on the abdominal aortic aneurysm (AAA) progression.

**Methods:**

ADSC-exos were isolated and identified. DiR and PKH67 staining were used to trace ADSC-exo in vivo and in vitro. Raw264.7 cells were applied to perform in vitro experiments, while a murine AAA model induced using angiotensin II (Ang II) was used for in vivo testing. The expression level of miR-17-5p in macrophages and Ang II-treated macrophages after ADSC-exos treatment was determined using reverse transcription-quantitative polymerase chain reaction (RT-qPCR). The target relation between miR-17-5p and thioredoxin-interacting protein (TXNIP) was identified by a dual-luciferase reporter gene assay. Artificial activation and block of experiments of miR-17-5p and TXNIP were conducted to clarify their functions in inflammation during AAA progression. The severity of AAA between groups was assessed by maximal aorta diameter, AAA incidence, survival rate, and histological stainings. Besides, inflammasome-related proteins and macrophage pyroptosis were further evaluated using western blot, RT-qPCR, and enzyme-linked immunosorbent assay (ELISA).

**Results:**

The ADSC-exos were isolated and identified. In vivo testing showed that ADSC-exos were mainly distributed in the liver. Meanwhile, in vitro experiments suggested that ADSC-derived exosomes were taken up by macrophages, while inside, ADSC-exos miR-17-5p decreased a TXNIP induced by Ang II by directly binding to its 3′-untranslated region (3’UTR). Furthermore, overexpression of miR-17-5p enhanced the therapeutic function of ADSC-exos on inflammation during AAA expansion in vivo, while its inhibition reversed this process. Finally, overexpressed TXNIP triggered macrophage pyroptosis and was alleviated by ADSC-derived exosomes in vitro.

**Conclusion:**

ADSC-exos miR-17-5p regulated AAA progression and inflammation via the TXNIP-NLRP3 signaling pathway, thus providing a novel insight in AAA treatment.

## Background

An abdominal aortic aneurysm (AAA) is a serious vascular disease characterized by segmental and permanent dilation of the abdominal aorta [1]. AAA can be dangerous if it is not spotted early on, as the rupture of an abdominal aortic aneurysm is usually lethal, with the fatality rate reaching up to 90% [2, 3]. With the development of open repair and endovascular aneurysm repair (EVAR) therapy, aneurysms larger than 5.5 cm (women > 4.5 cm) in diameter are effectively treated [4, 5]. However, currently, only 10% of patients are eligible for surgery, and there is no effective pharmacological treatment for patients who cannot undergo surgery [6].

AAA involves chronic inflammation of blood vessel walls. Thioredoxin-interacting protein (TXNIP) inflammasome-mediated inflammation in macrophages has an important role in various cardiovascular diseases, including AAA and atherosclerosis [7–9]. Usui et al. found that TXNIP-NOD-like receptor thermal protein domain associated protein 3 (NLRP3) inflammasome activation by mitochondrial oxidative stress in macrophages leads to the development of AAA, while the inhibition of NLRP3 can inhibit aneurysmal formation, arterial dilation rate, and inflammatory cell infiltration of ApoE^−/−^ mice induced by angiotensin II (Ang II) [10]. Furthermore, Xie et al. found that transplantation of adipose-derived mesenchymal stem cells (ADSCs) can significantly reduce inflammatory cell infiltration in AAA model mice through the paracrine approach and promote the M2 macrophage polarization thus protecting against AAA [11]. Nevertheless, the proteolysis and inflammatory microenvironment of the transplanted target tissue can directly affect the survival and migration of ADSC in the transplanted site, limiting its clinical application [12].

More recently, it was found that ADSC-derived exosomes (ADSC-exos) can decrease oxidative stress, activate PI3K/Akt pathway, and increase ATP levels by enhancing myocardial viability and preventing adverse remodeling after myocardial ischemia/reperfusion injury [13, 14]. Exosomes are 40–100 nm membranous microvesicles containing proteins similar to those derived from ADSCs and bioactive substances such as microRNAs (miRNAs), which can be exited by fusion with cell membranes and absorbed into target cells through membrane receptors or endocytosis [15]. Previous studies have discovered that exosomal miRNAs participate in the pathological process of AAA by modulating mRNAs translation [16]. For example, human mesenchymal stromal cell-derived extracellular vesicles can reduce aortic aneurysm formation and macrophage activation via miR-147 [17]. Besides, exosomal miR-106a has a pivotal role in abdominal aortic aneurysms by inducing vascular smooth muscle cell apoptosis [18]. Moreover, miR-17-5p participates in cardiovascular diseases like atherosclerosis, which is also found to decrease the thoracic aortic aneurysm [19, 20]. Also, a recent study found that TXNIP is a target gene for miR-17-5p [21, 22], and miR-17-5p can target TXNIP/NLRP3 pathway to suppress pancreatic β-cell pyroptosis in diabetic mice [23]. Furthermore, miR-17-5p facilitates the vascular repair of the aneurysm by regulating the PTEN-mediated PI3K/AKT/VEGFA pathway [20]. However, the role and underlying mechanisms of exosomal miR-17-5p in AAA are not fully understood. Hence, we hypothesized that ADSC-exos could inactivate TXNIP-NLRP3 inflammasome by delivering miR-17-5p to macrophages, thus inhibiting the progression of AAA.

In this current study, our objectives were to obtain the ADSC secreted exosomes; evaluate the distribution of the ADSC-exos in vivo and in vitro; and inject the ADSC-exos into the tail vein of AAA mice to demonstrate its therapeutic efficiency in vivo. Mechanistically, this study was further designed to explore the exosomal miR-17-5p of ADSCs in blocking TXNIP-NLRP3 inflammasome to acquire new strategy for the pharmaceutical treatment of AAA.

## Methods

### Animals

Six-week-old wild-type (WT) C57BL/6 mice and 8-week-old male ApoE^−/−^ C57BL/6 mice were purchased from Shanghai JieSiJie Laboratory Animals Co,. LTD (Shanghai, China). All the animals were housed in an environment with a temperature of 22 ± 1 ºC, relative humidity of 50 ± 1%, and a light/dark cycle of 12/12 h. All animal studies (including the mice euthanasia procedure) were done in compliance with the regulations and guidelines of Shanghai Ninth People’s Hospital, Shanghai Jiao Tong University School of Medicine institutional animal care and were conducted according to the AAALAC and the IACUC guidelines.

### Isolation and characterization of ADSCs

The subcutaneous adipose tissue was isolated sterile from the areas of groin of 6-week-old male WT C57/BL6 mice and digested by NB4 collagenase (#S1745403, Nordmark) for 1 h. After a filtration through 100 μm mesh, ADSCs were seeded overnight on culture plates with Dulbecco’s modified Eagle medium (DMEM) supplemented with 10% fetal bovine serum (FBS) and 100 U/ml penicillin/streptomycin in a humidified atmosphere containing 5%CO_2_/95% air at 37 °C. After the removal of suspended cells, the medium was replaced with a fresh one containing 5 ng/ml recombinant murine basic fibroblast growth factor (#450-33, Peprotech). The ADSCs of the third passage were collected and washed with phosphate-buffered saline (PBS). The phenotype of ADSCs was confirmed by flow cytometry using anti-mouse antibodies against CD29 (#102205, Biolegend), CD31 (#102405, Biolegend), CD34 (#110341-82, Invitrogen), CD45 (#157214, Biolegend), CD90 (#105305, Biolegend), and CD105 (#323203, Biolegend). Flow cytometry (Beckman Coulter, Fullerton, CA, USA) was performed as previously described [24]. In order to evaluate the multilineage differentiation of ADSCs, the third passage of ADSCs was incubated with adipogenic differentiation induction medium for 2 weeks and stained using oil-red-O, or incubated with osteogenic differentiation induction medium for 3 weeks and stained with alizarin red, respectively.

### miRNA transfection

A total of 1 × 10^6^ ADSCs were cultured in 10 mL of ADSC-conditioned medium overnight before miRNA transfection. ADSCs were then transfected with 10 nM of miR-17-5p inhibitor (RiboBio) and 10 nM of miRNA inhibitor negative control (NC) (RiboBio) by using Lipofectamine® 3000 (#L3000015, Thermo Fisher). To further investigate the role of miR-17-5p in the activation of TXNIP-NLRP3 inflammasome, ADSCs were transfected with miR-17-5p mimics (RiboBio) and 10 nM of miRNA mimics NC (RiboBio) using Lipofectamine® 3000 transfection reagent (#L3000015, Thermo Fisher). At 48 h post-transfection, ADSCs supernatant from each group was harvested for exosome isolation.

### Dual-luciferase reporter gene assay

To analyze common binding sites between miR-17-5p and TXNIP, a dual-luciferase reporter assay was performed according to the instructions (#E1910, Promega). Raw264.7 cells were cotransfected with a luciferase reporter carrying wild-type TXNIP (TXNIP-WT) or TXNIP mutated (TXNIP-MUT) of the binding site and miR-17-5p mimics or negative control. After 6 h, the medium was replaced with complete medium. At 48 h after transfection, the luciferase activities were detected with a dual-luciferase kit (Promega). Dual-luciferase ratios (reporter genes/internal control genes) were calculated, and then, the ratio differences among different groups were compared.

### Isolation and characterization of ADSC-exos

The extraction process of ADSC-exos was performed as previously described [25]. Briefly, the cell samples of ADSCs were subjected to successive centrifugations (300, 2000, and 10,000 g for 10 and 45 min, respectively), and the supernatant was collected. Subsequently, the garnered supernatant was ultracentrifuged at 110,000 × g for 75 min to obtain the precipitate that was resuspended in PBS and then subjected to centrifugation at 110,000 × g for 75 min again. Finally, the pellet was resuspended in PBS and identified by nanoparticle tracking analysis (NTA) (ZetaView, Particle Metrix, Meerbusch, Germany) to measure the diameter of the exosomes. Transmission electron microscopy (TEM) analysis (JEM-2100F, Japan Electronics, Tokyo, Japan) was used to evaluate the morphological characteristics of the exosomes. The exosome protein markers (CD63, CD81, and CD9) were determined by western blot.

### Co-culture of Raw264.7 cells with ADSC-exos

Raw264.7 cells were purchased from the Shanghai Cell Resource Center at the Institute of Life Sciences (Shanghai, China) and cultured with DMEM with 5% fetal bovine serum (FBS) (#10099141, Gibco) and 1% antibiotic/antimycotic solution (#15240062, Gibco) as previously described [26]. ADSC-exos were stained with PKH67 and were added to the medium of Raw264. After 24 h, fluorescence was detected using a fluorescence microscope (U-LH100HG, Olympus Corporation, Japan). In addition, we co-cultured Raw264.7 cells with exosomes derived from ADSC^vehicle^, ADSC^miR−17−5p inhibitor^, and ADSC^miR−17−5p mimics^. Then the expression levels of TXNIP, NLRP3, IL-18, IL-1β, and cleaved caspase-1 were investigated by RT-qPCR and western blot. Moreover, the level of miR-17-5p in Raw264.7 cells of different groups was detected by RT-qPCR.

### SiRNA and plasmid transfection

SiRNA against TXNIP and scrambled siRNA were purchased from RiboBio, and TXNIP overexpression plasmid and control plasmid were constructed by GeneChem. To determine the role of TXNIP in inflammasome activation in macrophages, siRNA and plasmid were transfected into RAW264.7 cells using Lipofectamine® 3000 (#L3000015, Thermo Fisher) according to the manufacturer's instructions. At 24 h post-transfection, the effects of gene silencing and overexpression were measured by western blot. Inflammasome activation in these cells by Ang II (10 µM, 12 h) (#A9525, Sigma) treatment was then evaluated by western blot analysis and enzyme-linked immunosorbent assay [27, 28].

### In vivo tracing of ADSC-exos and exosomes injection

To label ADSC-exos in vivo, 1,1’-dioctadecyltetramethyl indotricarbocyanine iodide (DiR) (#22070, Beijing Fluorescence Biotechnology Co. Ltd) was dissolved in dimethylsulfoxide (DMSO) at a concentration of 1 mg/ml. Next, DiR solution was mixed with ADSC-exos at a ratio of 2 μg DiR/100 μg ADSC-exos in PBS for 1 h and ultracentrifugated (100,000 g for 1 h) to exclude dissociative DiR and DMSO. The pellet was then resuspended in PBS at a concentration of 1 μg exosome/1μL PBS. Subsequently, PBS, DiR, and DiR-exosome were, respectively, injected via the tail vein of mice. After 72 h, in vivo fluorescence imaging machine (VISQUE In vivo Smart-LF, Vieworks, Korea) was used for fluorescence intensity analysis. ADSC-exos were then injected 100 μg per day every three days via tail vein according to the literature for a total of 28 days [29]. Specifically, we treated the Ang II-induced AAA mice with exosomes derived from ADSC^vehicle^, ADSC^miR−17−5p inhibitor^, and ADSC^miR−17−5p mimics^. Then AAA tissue and blood samples were collected and utilized for the following experiments.

### Construction of AAA mice model

A 12-week-old male ApoE^−/−^ C57BL/6 mice (Shanghai Research Center for Model Organism, China) fed with a high-fat diet (HFD) (#D12492, ResearchDiets, USA) were used for AAA model construction. Firstly, the mice were anesthetized with the intraperitoneal injection of 0.3 ml/kg of 0.6% amobarbital. Next, osmotic minipumps (#Alzet Model 2004, Charles River Laboratories, Inc) were subcutaneously implanted in the lateral back of mice to infuse Ang II (#A9525; Sigma) at a rate of 1550 ng/kg per minute or saline. Finally, the mice were fed with HFD for 28 days.

To further validate the role of ADSC-exos in inflammasome activation, primary murine macrophages were generated from bone marrow harvested from AAA mice and cultured as previously described [30].

### Cytokines detection by enzyme-linked immunosorbent assay

The cytokines concentration in cell supernatant or serum was detected using ELISA kits following the manufacturer's instructions: Mouse IL-18 ELISA kit (#ab216165, Abcam) and Mouse IL-1β ELISA kit (#KE10003, Proteintech).

### Histological analysis

On day 28, mice were euthanized with the intraperitoneal injection of 0.3 ml/kg of 0.6% amobarbital and perfused with 4% paraformaldehyde (PFA) through the left cardiac apex. The entire aorta was dissected, fixed in 4% PFA, embedded in paraffin, and sectioned. The H&E, Verhoeff’s Van Gieson (EVG), Masson trichrome, and immunofluorescence stainings were performed by Shanghai Runnerbio Technology CO. Ltd (Shanghai China) according to standard procedures. For the immunofluorescence staining of the muscle sections, the F4/80 (#28463-1-AP, Proteintech) were stained with FITC (Abcam); the TXNIP (#ab188865, Abcam) was stained with cy3 (Abcam), and the nucleus was stained with DAPI (Abcam). Image J software (Rawak Software, Inc. Germany) was used for quantitative analysis of elastin degradation and immunofluorescence intensity as previously described [31].

### Western blot

Total cell or tissue extracts were lysed with RIPA peptide lysis buffer containing 1% protease inhibitors (Roche; #11836153001). After being sealed with 5% bovine serum albumin (BSA) (#B2064, Sigma), the membranes were incubated with the following antibodies: anti‐TXNIP, anti‐NLRP3, anti-caspase-1, anti‐IL‐1β, anti‐IL‐18, and anti‐GAPDH (glyceraldehyde‐3phosphate dehydrogenase) at 37 °C overnight. The membranes were then rinsed three times with tris-buffered saline tween (TBST) and incubated with peroxidase‐labeled secondary antibodies at room temperature for 2 h. The item number information for antibodies is listed in Table 1. Finally, the protein signals were developed using an enhanced chemiluminescence (ECL) reagent (#GERPN2106, Sigma) and visualized with a Biorad Gel Doc EQ system. The gray value was quantified using Image J software (Rawak Software, Inc. Germany).

**Table 1:** Antibodies used in western blot

Antibodies	Cat. No. & Company	Dilution ratio
TXNIP	ab188865, Abcam	1:1000
NLRP3	ab263899, Abcam	1:1000
Cleaved-caspase-1	ab179515, Abcam	1:1000
GSDMD	ab209845, Abcam	1:1000
IL-18	#57,058, CST	1:1000
IL-1β	#31,202, CST	1:1000
CD63	ab134045, Abcam	1:1000
CD81	#56,039, CST	1:1000
CD9	#13,403, CST	1:1000
GAPDH	#5174, CST	1:20,000
Anti-mouse IgG (HRP)	#7076, CST	1:5000
Anti-rabbit IgG (HRP)	#7074, CST	1:5000

*TXNIP* thioredoxin-interacting protein; *NLRP3* NOD-like receptor thermal protein domain associated protein 3; *GSDMD* Gasdermin-D; *GAPDH* glyceraldehyde-3-phosphate dehydrogenase; *Abcam* Abcam Inc., Cambridge, MA, USA; *CST* Cell Signaling Technology, Danvers, MA, USA

### Reverse transcription-quantitative polymerase chain reaction (RT-qPCR)

Total RNA of each sample was extracted from the cells and mouse AAA tissue using TRIzol reagent (#155960-18, Invitrogen). Then, the extracted RNA was reverse transcribed into cDNAs using a kit according to instructions (#AT341, TransGen Biotech). The cDNA template amplification was conducted by RT-qPCR using the AceQ Universal SYBR qPCR Master Mix (#Q511-03, Vazyme). A U6 transcript was used for the normalization of miR-17-5p expression; the relative mRNA expression of the genes was normalized by GAPDH. The primer sequences listed in Table 2 were synthesized by Shanghai Sangon Biotech Co., Ltd. (Shanghai, China). RT-qPCR of each sample was repeated in triplicate, and the relative expression fold change of the genes was calculated by 2^‐ CΔΔ t^ method.

**Table 2:** Primer nucleotide sequences of RT-qPCR

Name	Primer sequence
m-miR-17-5p	CAAAGTGCTTACAGTGCAGGTAG
U6-F	CTCGCTTCGGCAGCACA
U6-R	AACGCTTCACGAATTTGCGT
m-TXNIP-F	TCAATACCCCTGACCTAATGGC
m-TXNIP-R	TTCTGTCAATTCGAGCAGAGAC
m-NLRP3-F	ATTACCCGCCCGAGAAAGG
m-NLRP3-R	TCGCAGCAAAGATCCACACAG
m-IL-18-F	GACTCTTGCGTCAACTTCAAGG
m-IL-18-R	CAGGCTGTCTTTTGTCAACGA
m-IL-1β-F	GAAATGCCACCTTTTGACAGTG
m-IL-1β-R	TGGATGCTCTCATCAGGACAG
m-GAPDH-F	TGGCCTTCCGTGTTCCTAC
m-GAPDH-R	GAGTTGCTGTTGAAGTCGCA

*RT-qPCR* reverse transcription-quantitative polymerase chain reaction; *F* forward; *R* reverse; *TXNIP* thioredoxin-interacting protein; *NLRP3* NOD-like receptor thermal protein domain associated protein 3; *GAPDH* glyceraldehyde-3-phosphate dehydrogenase

### Statistical analysis

GraphPad Prism version 8.0 (GraphPad, La Jolla, CA, USA, http://www.graphpad.com) was applied for data analysis. The mean ± standard deviation was used to describe parametric values. The Chi-square test was conducted to evaluate the survival rate. The one-way analysis of variance (ANOVA) and two-tailed Student’s t-test were performed to compare data between groups. A *P* value < 0.05 was considered statistically significant.

## Results

### Isolation and characterization of ADSCs and ADSC-exos

**Fig. 1:**
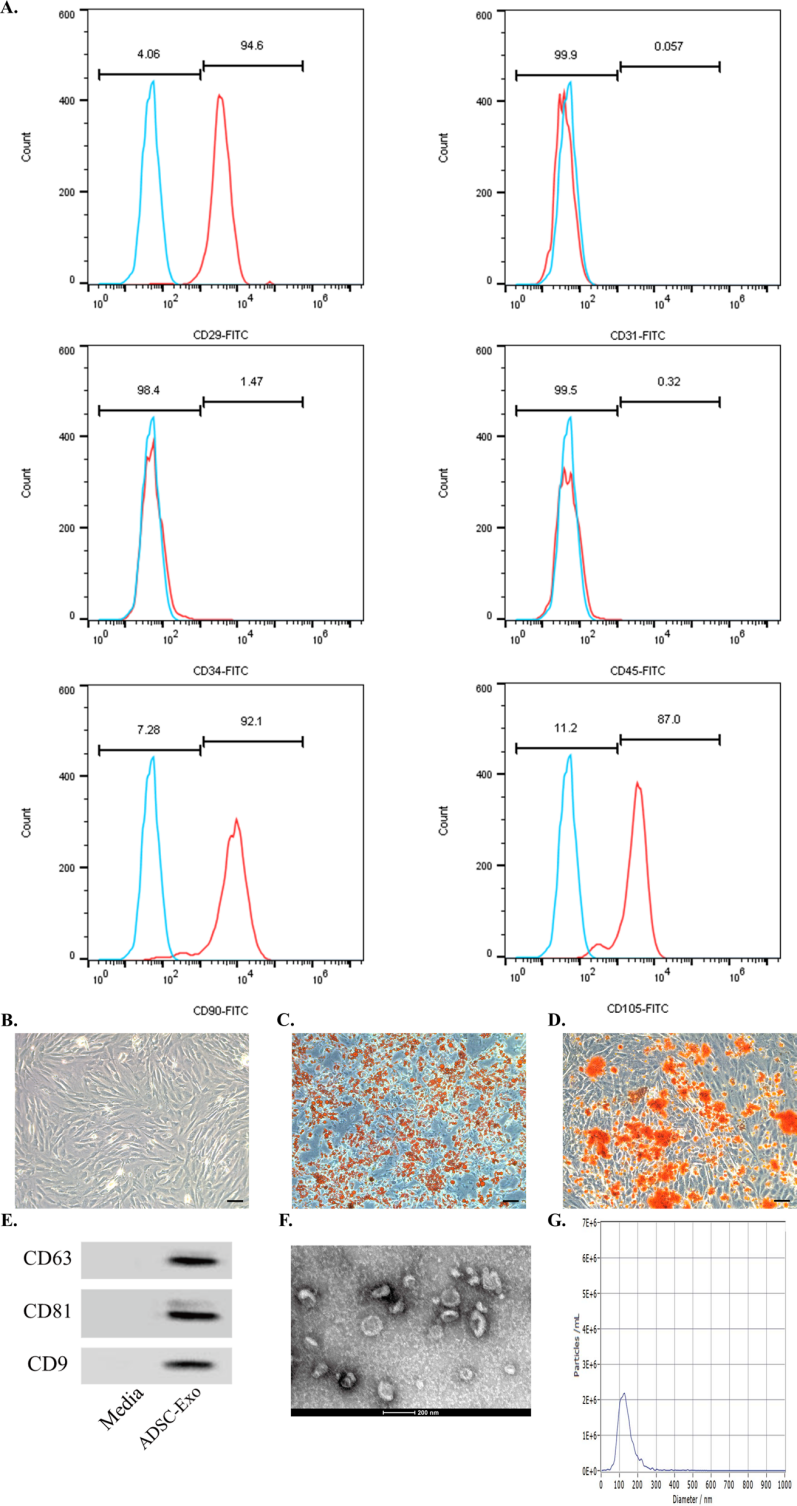
Isolation and characterization of ADSCs and ADSC-exos. **A** ADSC-specific surface markers such as CD29, CD31, CD34, CD45, CD90, and CD105 measured by flow cytometry. **B** P3-ADSCs in vitro showed typical spindle fibroblast-like morphology. Scale bar: 100 μm. **C** Positive Oil-Red-O staining of ADSCs was assessed after adipogenic differentiation induction for 2 weeks. Scale bar: 100 μm. **D** Positive Alcian Blue staining of ADSCs assessed after osteogenic differentiation induction for 3 weeks. Scale bar: 100 μm. **E** Western blot of exosomal biomarkers. **F**, **G** The ADSC-derived exosomes showed a circular disk shape under TEM (F) with the average particle size at 100 nm according to NTA analysis (G). ADSC, Adipose-derived mesenchymal stem cell; P3, passage 3

ADSCs were collected from the inguinal region of mice and identified using flow cytometry. The ADSC-specific biomarkers such as CD29, CD31, CD34, CD45, CD90, and CD105 were positively expressed (Fig. 1A). The p3 ADSCs presented characteristics as typical spindle fibroblast-like cells (Fig. 1B).

Next, positive oil-red-O or alizarin red staining of ADSCs was assessed after adipogenic differentiation induction for 2 weeks (Fig. 1C) or osteogenic differentiation induction for 3 weeks (Fig. 1D).

Western blot analysis of ADSC-exos further suggested positive expression of exosomal markers such as CD63, CD81, and CD9 (Fig. 1E). Thereafter, exosomes were generated from ADSCs supernatant by ultracentrifugation, showing a circle shape; an average particle size was 100 nm based on TEM observation and NTA (Fig. 1F,G).

### Tracing of ADSC-exos in vivo and in vitro

**Fig. 2:**
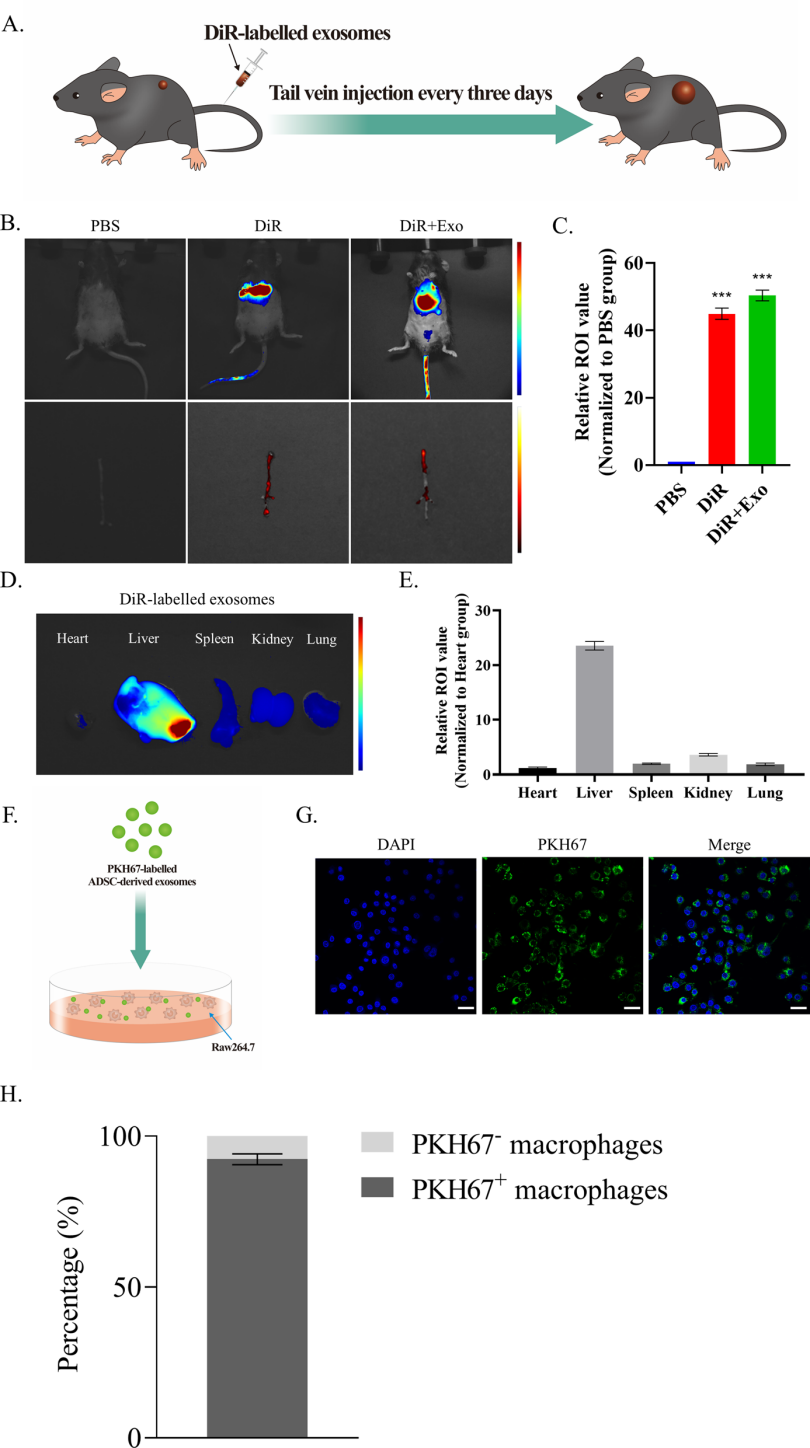
Tracing of ADSC-derived exosomes in vivo and in vitro. **A** DiR-labeled ADSC-derived exosomes injected into mouse tail veins. **B**, **C** Fluorescence imaging results of mice and dissected aortas (**B**), and statistical analysis (**C**). **D**–**E** Representative fluorescence image of tissues from the DiR-labeled exosomes-treated group on day 3 (**D**), and statistical analysis (**E**). **F** The PKH67-labeled exosomes were incubated with macrophages. **G**–**H** The PKH67 labeled ADSC-exos taken up by the macrophages were examined under the fluorescence microscope (**G**), and statistical analysis (**H**). Scale bar: 25 μm. Repetition = 3. A one-way ANOVA was performed to compare data between groups. *P value < 0.05, **P value < 0.01, ***P value < 0.001

DiR-labeled ADSC-derived exosomes were injected into mouse tail veins to track the distribution in mice (Fig. 2A). The results showed that the fluorescence was mainly distributed in the liver (Fig. 2B), suggesting the ADSC-derived exosomes were well metabolized through the liver. The fluorescence intensity of DiR-labeled ADSC-exos was higher than that of the DiR group (*P* < 0.05) (Fig. 2C). Furthermore, the ex vivo influorescence images showed an obvious accumulation of the ADSC-exos signal in the liver while the signal was also observed in the kidney, lung, and spleen (Fig. 2D,E).

Next, we examined whether ADSC-exos could be taken up by Raw264.7 cells by labeling the exosomes with PKH67. Then PKH67-labeled exosomes were incubated with Raw264.7 cells (Fig. 2F). In addition, a confocal microscopy analysis showed that the labeled ADSC-derived exosomes were taken up by the macrophages (Fig. 2G), and the corresponding ratio of PKH67 positive macrophages was presented (Fig. 2H).

### ADSC-exos can antagonize Ang II-induced AAA and TXNIP-NLRP3 inflammasome activation in macrophages

**Fig. 3:**
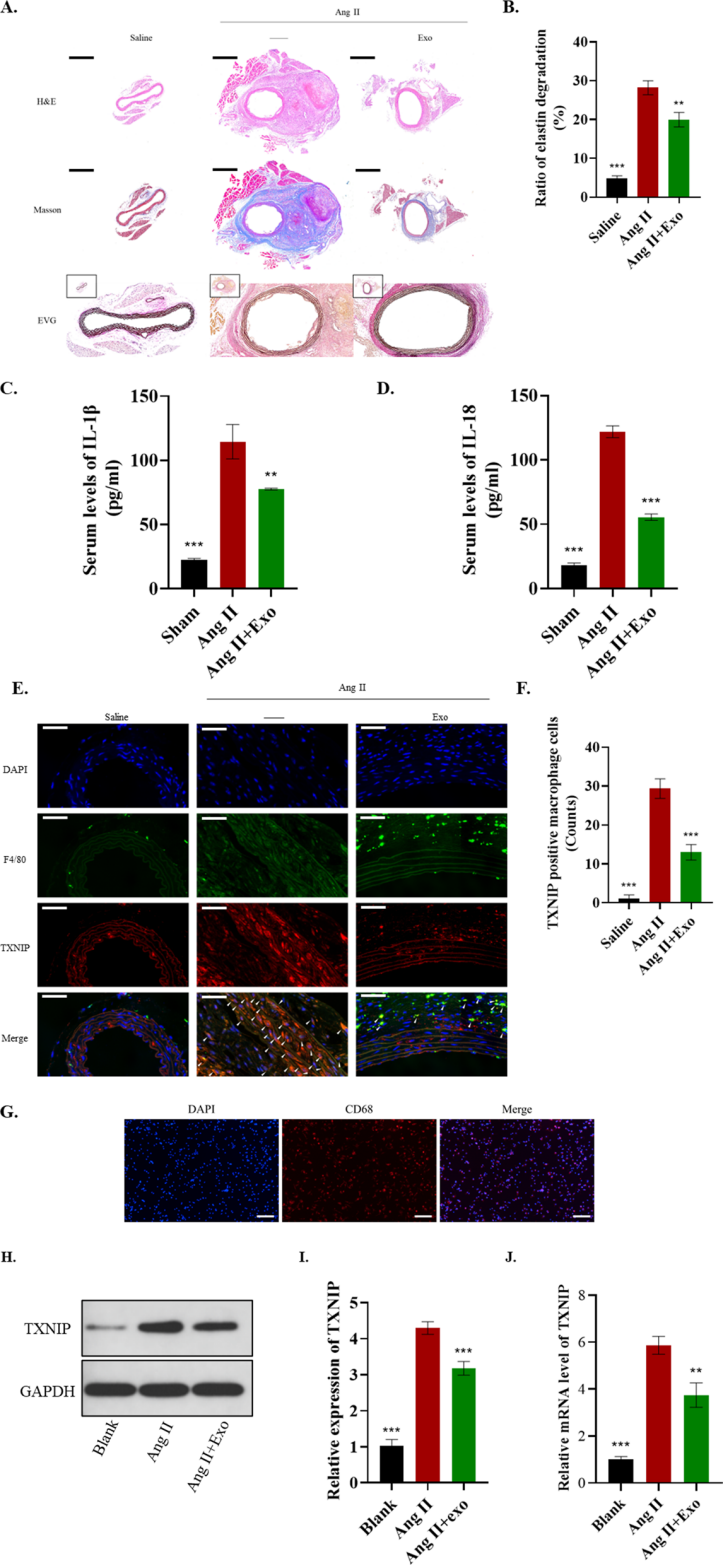
ADSC-exo inhibited AAA progression and TXNIP-NLRP3 inflammasome activation in macrophages. **A**, **B** The H&E, Masson trichrome and EVG staining (**A**) and statistical analysis (**B**) of elastin degradation in mice. Scale bar: 600 μm. **C**–**D** Serum levels of IL-1β and IL-18. **E**–**F** Immunofluorescence analysis of TXNIP positive macrophages in AAA mice. Scale bar: 50 μm. **G** Identification of the primary murine macrophages by CD68. Scale bar: 100 μm. **H**, **I** Western blot and statistical analysis. **J** RT-qPCR analysis. Repetition = 3. A one-way ANOVA were performed to compare data between groups. *P value < 0.05, **P value < 0.01, ***P value < 0.001

To determine the effect of ADSC-exos on the pathological process of AAA, we injected ADSC-exos into the tail vein of AAA mice for 28 days. The Masson trichrome staining indicated that the enhanced elastin degradation induced by Ang-II was reduced in the exosome injection group (*P* < 0.05) (Fig. 3A,B). Also, the elevation of serum IL-1β and IL-18 levels were alleviated by ADSC-exos treatment (*P* < 0.05) (Fig. 3C,D). In addition, there were fewer TXNIP positive macrophages in the area of AAA in mice treated with ADSC-exos (*P* < 0.05) (Fig. 3E,F). Besides, the primary murine macrophages were isolated and identified using CD68 (Fig. 3G). The upregulated level of TXNIP in primary murine macrophages was reduced by ADSC-exos (*P* < 0.05) (Fig. 3H–J). The data suggest that ADSC-exos can antagonize Ang II-induced AAA and TXNIP-NLRP3 inflammasome activation in macrophages**.**

### ADSC-exos ameliorated inflammation in Ang II-pretreated Raw264.7 cells by modulating TXNIP

**Fig. 4:**
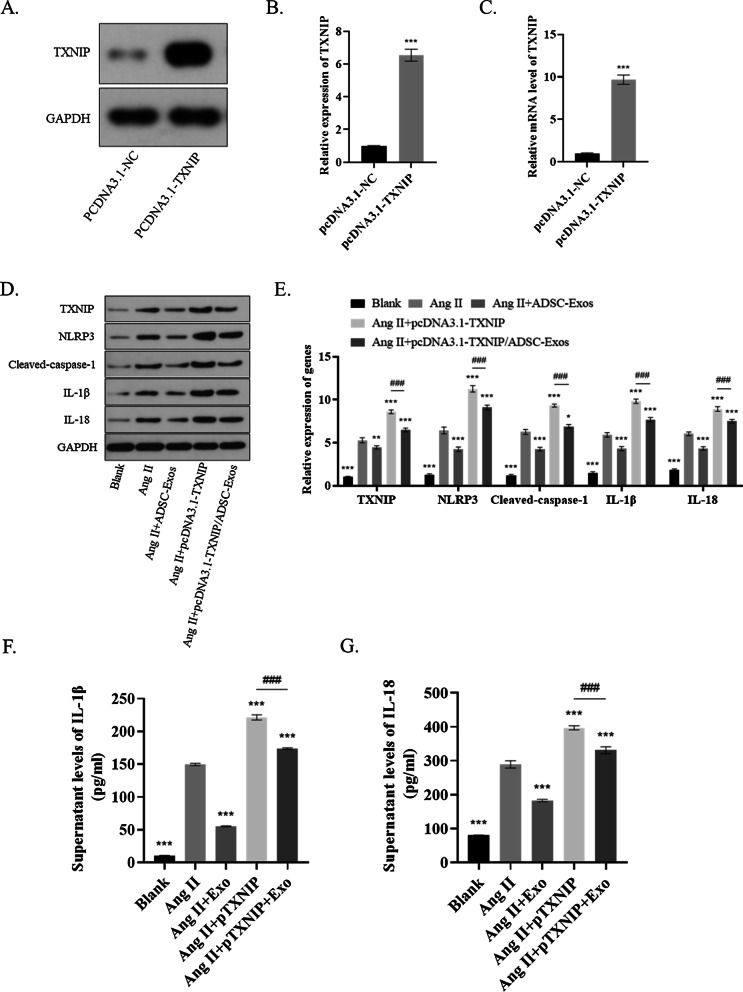
ADSC-exo ameliorated inflammation in Ang II-pretreated Raw264.7 by modulating TXNIP. **A**–**C** Western blot and RT-qPCR analyses were performed to evaluate the level of an overexpressed TXNIP in Raw264.7 cells. **D**–**E** ADSC-exos reduced the inflammasome-related gene expression of Ang II-treated Raw264.7 cells, while gain of TXNIP counteracted such inhibitory effects of ADSC-exos. **F**–**G** ELISA analysis of IL-18 and IL-1β. Repetition = 3. The one-way ANOVA and two-tailed Student’s t-test were performed to compare data between groups. *P value < 0.05, **P value < 0.01, ***P value < 0.001

In vivo demonstrated that ADSC-derived exosome can inhibit inflammasome activation, thus inhibiting aneurysm inflammation. Subsequently, in vitro experiments were carried out in Raw264 cells to further verify whether ADSC-exo exerted protective effects against AAA formation, mainly through TXNIP. An overexpressed TXNIP in Raw264.7 cells was established by transfecting pcDNA3.1-TXNIP into the cells (*P* < 0.05) (Fig. 4A–C). ADSC-exo reduced the inflammasome-related gene expression of Ang II-treated Raw264.7 cells, including TXNIP, NLRP3, cleaved-caspase-1, IL-18, and IL-1β. Such inhibitory effects were significantly counteracted by TXNIP overexpression (*P* < 0.05) (Fig. 4D–E). The levels of IL-18 and IL-1β in the cellular supernatant were consistent with the results above (*P* < 0.05) (Fig. 4F–G).

### ADSC-exo carried miR-17-5p into macrophages to target TXNIP

**Fig. 5:**
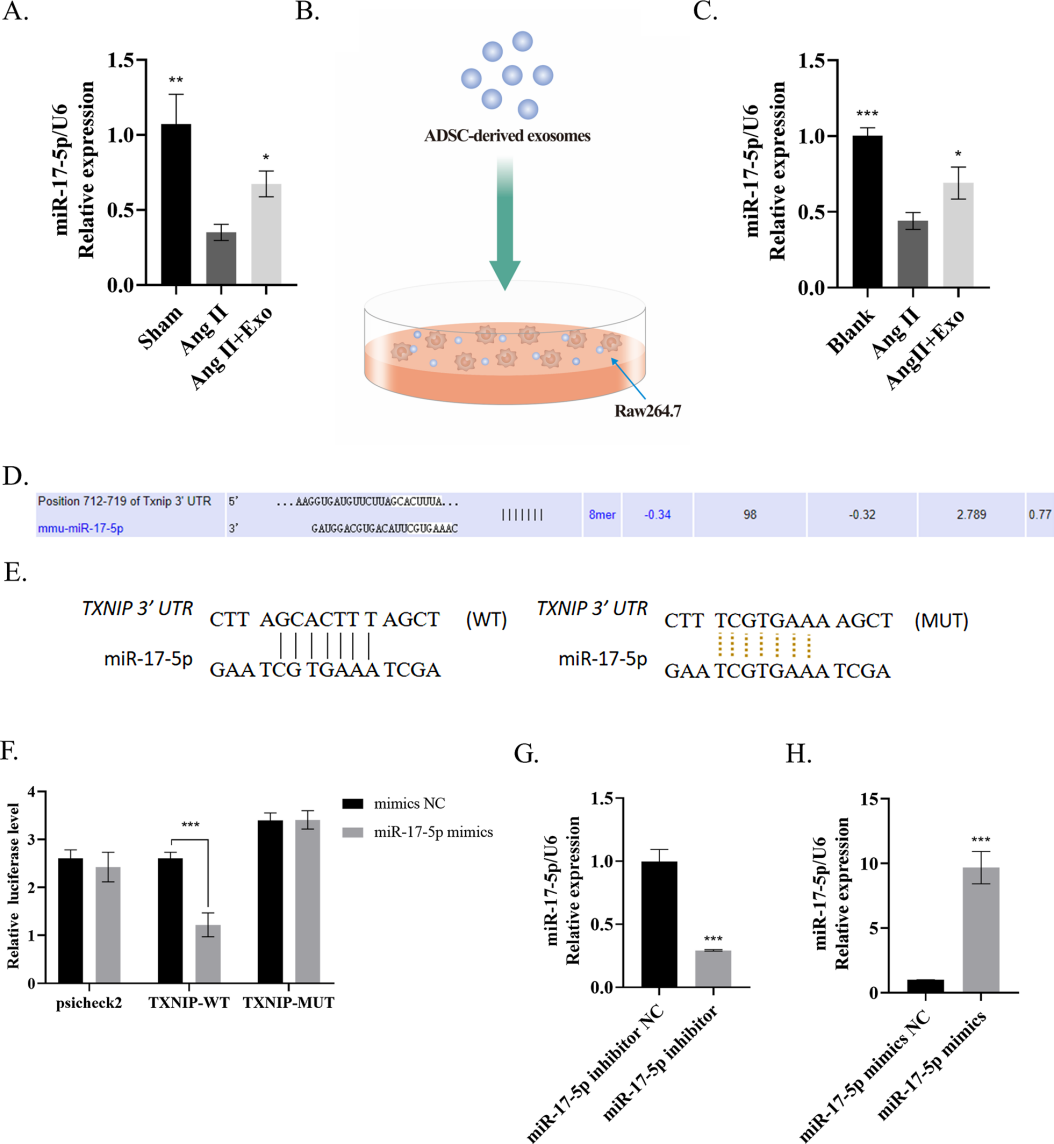
ADSC-exos carried miR-17-5p into macrophages to target TXNIP. **A** MiR-17-5p decreased in AAA tissue and was upregulated by ADSC-derived exosomes injection. **B**, **C** The Raw264.7 cells were co-cultured with ADSC-exos overnight (**B**), and the miR-17-5p level was detected by RT-qPCR (**C**). **D** Target relation between miR-17-5p and TXNIP predicted on https://www.targetscan.org. **E**, **F** Target relation of miR-17-5p and TXNIP identified using dual-luciferase reporter gene assay. **G**, **H** RT-qPCR was conducted to evaluate the levels of miR-17-5p. Repetition = 3; The one-way ANOVA and two-tailed Student’s t-test were performed to compare data between groups. *P value < 0.05, **P value < 0.01, ***P value < 0.001

We discovered that miR-17-5p was decreased in AAA tissue; this process could be reversed (normalized) when using exosomes (*P* < 0.05) (Fig. 5A). Then ADSC-exos were added into the medium of Raw264.7 cells for 12 h (Fig. 5B). Consistent results were observed in Raw264.7 cells (*P* < 0.05) (Fig. 5C). It has been experimentally validated that TXNIP is a target gene for miR-17-5p [21, 22]. According to the prediction of the database (https://www.targetscan.org), miR-17-5p binds directly to the 3’UTR of TXNIP (Fig. 5D). Next, the target relation of miR-17-5p and TXNIP was further clarified using a dual-luciferase reporter gene assay (*P* < 0.05) (Fig. 5E,F).

Furthermore, in order to investigate whether the inhibitory effect of ADSC-exos on TXNIP inflammasome was caused by miR-17-5p, we transfected ADSCs with miR-17-5p inhibitor or miRNA inhibitor NC for producing ADSC-exo^miR−17−5p inhibitor^ and ADSC-exo^miR−17−5p inhibitor NC^, respectively (Fig. 5G). Simultaneously, ADSCs were also transfected with miR-17-5p mimics and miRNA mimics NC to construct ADSC-exo^miR−17−5p mimics^ and AMSC-Exo^miR−17−5p mimics NC^, respectively (Fig. 5H).

### Exosomal miR-17-5p from ADSC-derived exosomes can suppress the AAA progression

**Fig. 6:**
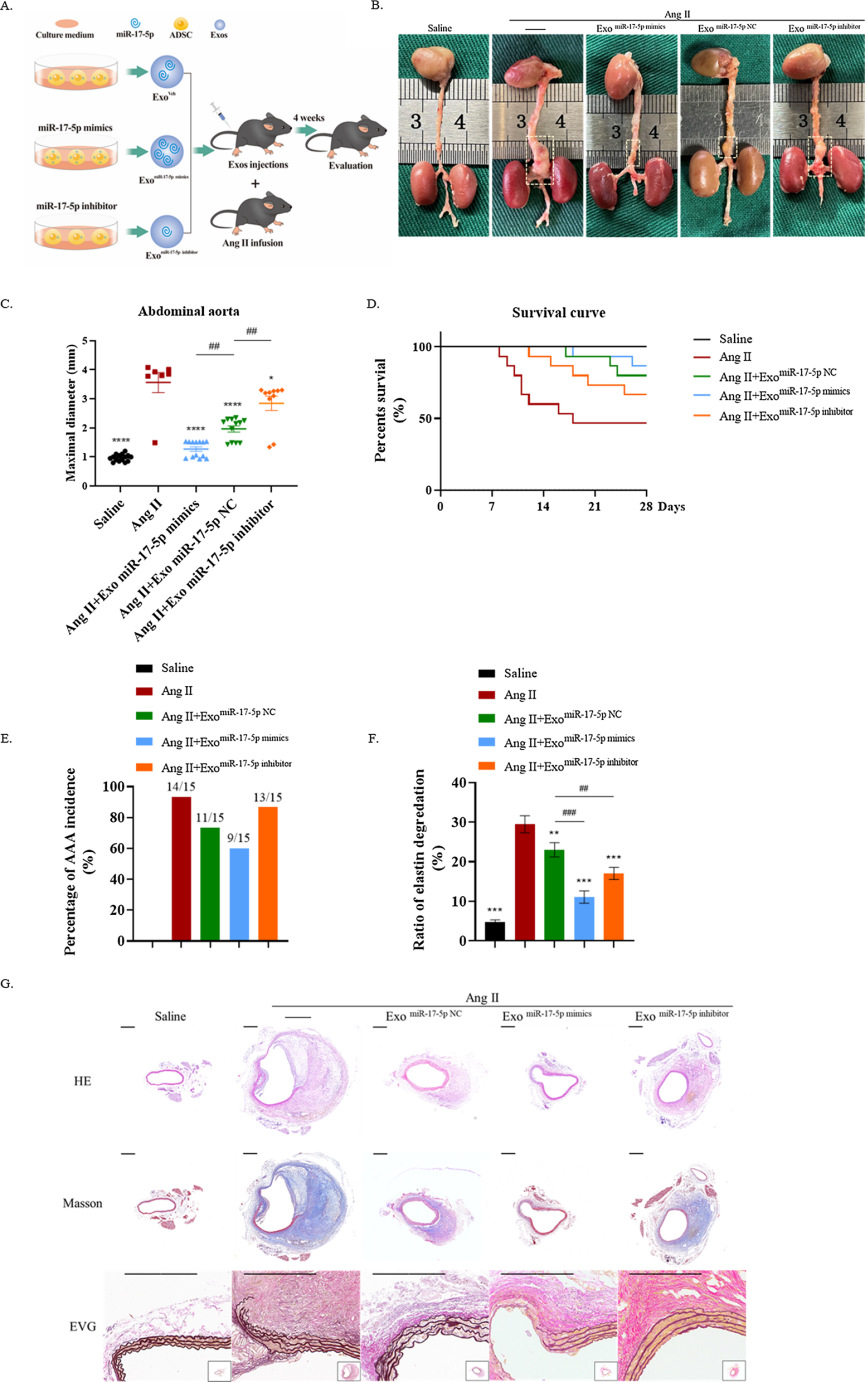
Exosomal miR-17-5p from ADSC-derived exosomes suppressed the AAA progression. **A** Schematic flow diagram of injections of exosomes gain or loss miR-17-5p in AAA mice. **B** Representative images of AAA. **C** Maximal diameter of the abdominal aorta. **D** AAA incidence. **E** Survival curve. **F** Statistical analysis of elastin degradation, repetition = 3. **G** Representative H&E, Masson trichrome and EVG staining of the mice aorta. Scale bar: 600 μm. In B through E, n = 15 for five different groups. The one-way ANOVA was performed to compare data between groups. *P value < 0.05, **P value < 0.01, ***P value < 0.001

Next, we examined the role of exosomal miR-17-5p on AAA progression in vivo. Briefly, mice were randomly divided into 5 groups; each group received a different concentration of exosomal miR-17-5p in AAA (Fig. 6A). ADSC-exos treatment significantly reduced both the maximal diameter of the abdominal aorta (1.97 ± 0.36 mm) and the incidence of the AAA (73.33%) in comparison with model mice (maximal diameter of 3.56 ± 0.85 mm and AAA incidence of 93.33%. Additionally, such therapeutic effect was enhanced in the exosome miR-17-5p mimics group (maximal diameter of 1.27 ± 0.27 mm and AAA incidence of 60%, while exosomal miR-17-5p knockdown led to the opposite trend (Fig. 6B,C,E). Consistently, we also observed difference in the AAA incidence between Exo^miR−17−5p inhibitor^ mice (86.67%) and Exo^miR−17−5p NC^ mice. Due to the incidence of aneurysms and rupture in this model, we observed that ADSC-exos injection significantly improved the survival rate (80%) compared to that in Ang II mice (46.67%) (P = 0.0304). Among the other groups, death occurred on day 12 in the Exo^miR−17−5p inhibitor^ group but was delayed for 5 days in the Exo^miR−17−5p NC^ group, emphasizing that the treatment effect of the Exo^miR−17−5p inhibitor^ group was worse than that of the Exo^miR−17−5p NC^ group. Compared with Exo^miR−17−5p NC^ mice, Exo^miR−17−5p mimics^ mice died on day 18, which also presented a decreasing mortality rate (Fig. 6D).

Elastin degradation is critical for developing AAA [32]. We comprehensively evaluated the elastin degradation in the mouse AAA model by combining EVG with Masson trichrome staining (Fig. 6G). The histological analysis was consistent with the above results (*P* < 0.05) (Fig. 6F).

### ADSC-exo-carried miR-17-5p inhibits TXNIP-NLRP3 inflammasome activation in vivo

**Fig. 7:**
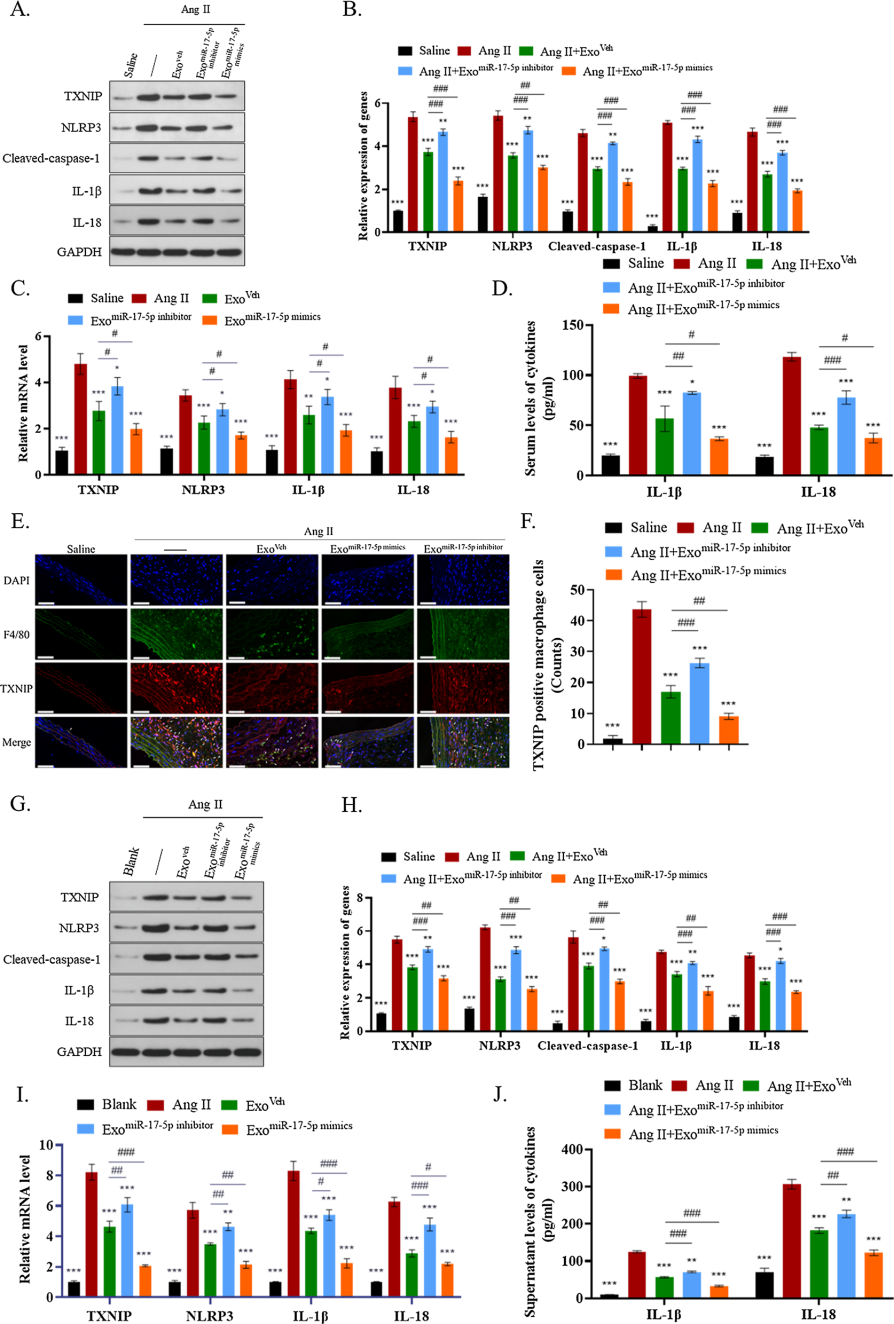
ADSC-exo-carried miR-17-5p inactivated TXNIP-NLRP3 inflammasome. **A**–**C** The protein levels of TXNIP, NLRP3, cleaved-caspase-1, IL-18, and IL-1β in Ang II-induced AAA determined using western blot and RT-qPCR. **D** ELISA analysis of IL-18 and IL-1β in mice serum. **E**–**F** Immunofluorescence analysis of TXNIP positive macrophages in different groups**.** Scale bar: 50 μm. **G**–**I** The protein levels of TXNIP, NLRP3, cleaved-caspase-1, IL-18, and IL-1β in Ang II-treated Raw264.7 cells were evaluated by western blot and RT-qPCR analyses. **J** ELISA analysis of IL-18 and IL-1β in the supernatant of Raw264.7 cells. Repetition = 3. The one-way ANOVA was performed to compare data between groups. *P value < 0.05, **P value < 0.01, ***P value < 0.001

To determine whether ADSC-exos-carried miR-17-5p inactivated TXNIP-NLRP3 inflammasome, we measured the inflammasome-related mRNA and protein levels in these five mice groups. The results suggested that the protein levels of TXNIP, NLRP3, cleaved-caspase-1, IL-18, and IL-1β were significantly enhanced in Ang II-treated AAA, and their levels were inhibited by ADSC-exos, especially in those treated with Exo^miR−17−5p mimics^. Meanwhile, miR-17-5p knockdown impaired the negative regulatory effect of ADSC-exos on TXNIP inflammasome, which presented a relative increasing trend compared with the Exo^miR−17−5p Veh^ group (*P* < 0.05) (Fig. 7A–C). The serum levels of IL-1β and IL-18 were in accordance with the findings mentioned above (*P* < 0.05) (Fig. 7D).

TXNIP-NLRP3 inflammasome-mediated inflammation in macrophages has an important role in various cardiovascular diseases, including AAA. Immunofluorescence analysis revealed that the TXNIP expression in F4/80 labeled macrophages in AAA of each group supported the results above (*P* < 0.05) (Fig. 7E,F). Then, Raw264.7 cells were treated with seven different groups of ADSC-exos, including two negative control groups, to conduct in vitro validation experiments, and the same results of the AAA mice model were reproduced (*P* < 0.05) (Fig. 7G–J).

### ADSC-derived exosomes blocks TXNIP-mediated cell pyroptosis of macrophages in inflammation induced by Ang II

**Fig. 8:**
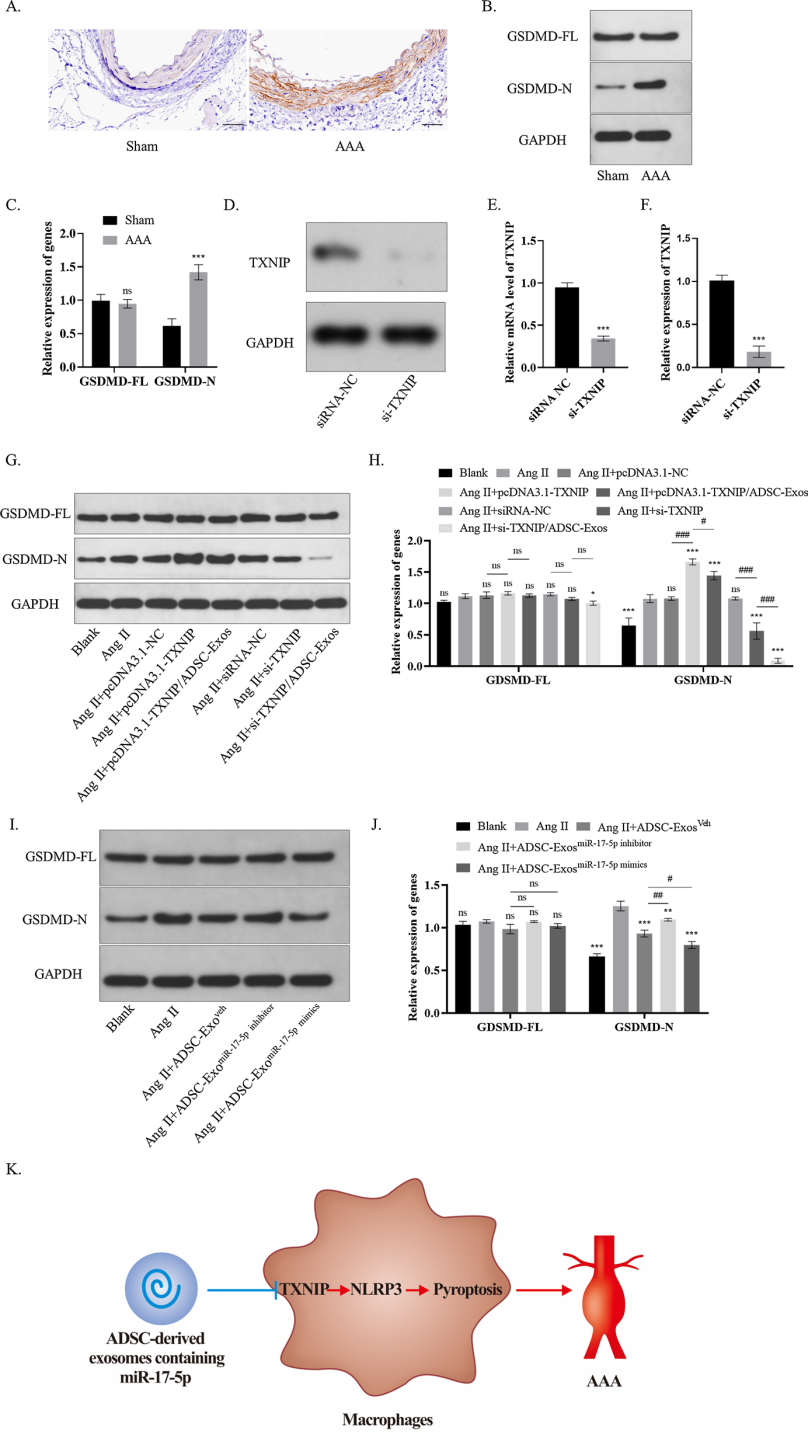
ADSC-derived exosomes blocked TXNIP-mediated cell pyroptosis of macrophages in inflammation induced by Ang II. **A** Positive GSDMD cells in Ang II-induced AAA mice detected using immunohistochemistry. Scale bar: 50 μm. **B**, **C** The level of cleaved GSDMD-N after Ang II treatment detected using western blot analysis **D-F** The level of TXNIP knockdown was validated by western blot (D-E) and RT-qPCR analysis (**F**). **G**–**H** GSDMD expression in Raw264.7 cells receiving different treatments was evaluated using western blot analysis. **I**–**J** Western blot analysis showed the treatment efficacy of exosomal miR-17-5p mimics versus exosomal miR-17-5p inhibitor on GSDMD expression in Raw264.7 cells. **K** Proposed model for exosomal miR-17-5p from ADSC as a therapeutical strategy of AAA. Repetition = 3; The one-way ANOVA and two-tailed Student’s t-test were performed to compare data between groups. *P value < 0.05, **P value < 0.01, ***P value < 0.001

Previous studies have suggested that TXNIP regulates the activation of cell pyroptosis, thus promoting the release of cytokines like IL-18 and IL-1β [33]. In this study, the elevated expression of the positive rate of gasdermin D (GSDMD), a key molecule in the cell pyroptosis signaling, was observed in AAA tissue (Fig. 8A). Western blot analysis also elucidated that the cleaved GSDMD-N upregulated in AAA tissue (Fig. 8B,C) We have established an overexpressed TXNIP in Raw264.7 cells as mentioned above, a TXNIP siRNA plasmid was also transduced into Raw264.7 cells. Then, the mRNA level of TXNIP knockdown was validated by western blot (Fig. 8D,E) and RT-qPCR analysis (Fig. 8F). Subsequently, the Raw264.7 cells in this group were subsequently co-cultured with ADSC-exos after being treated with Ang II, and then the GSDMD-N was significantly decreased by ADSC-exos. In addition, the Raw264.7 cells in the knockdown group presented a drop in GSDMD-N. The decline of GSDMD-N was even more significant after ADSC-exos were added (Fig. 8G,H). Moreover, the elevated expression of cleaved GSDMD-N induced by Ang II was decreased by ADSC-exos, which presented an enhanced efficacy in ADSC-exos^miR−17−5p mimics^ group. Meanwhile, miR-17-5p knockdown weakened the inhibitory effect of ADSC-exos on cleaved GSDMD-N (*P* < 0.05) (Fig. 8I,J). These results further identified that TXNIP triggered macrophage pyroptosis in the inflammation during AAA, which can be suppressed by exosomal miR-17-5p.

## Discussion

Studies have suggested that inflammation has a critical role in the pathological process of AAA and that ADSC-exos may be used to treat inflammation-related vascular diseases [34, 35]. For instance, we have reported that exosomes from adipose mesenchymal stem cells overexpressing stanniocalcin-1 promoted reendothelialization after carotid endarterium mechanical injury [36]. Nevertheless, the role and underlying mechanisms of ADSC-exos in AAA progress remain poorly investigated. In the present study, we provided novel insights into the effect of ADSC-derived exosomes in modulating the AAA pathological process.

TXNIP exists as a ubiquitously expressed protein activated by various cellular stresses, such as inflammation and oxidative stress [37]. TXNIP can accelerate the process of releasing inflammatory cytokines. Current findings reveal that TXNIP inflammasome participates in the pathogenesis of cardiovascular diseases triggered by inflammation [38, 39]. However, most investigations on the TXNIP inflammasome in AAA focus on the vascular smooth muscle cells.

Previous studies, including our research, have demonstrated the therapeutic potential of exosomes in vascular diseases, including diabetic limb ischemia, carotid endarterium injury, atherosclerosis, and abdominal aortic aneurysm [36, 40, 41]. In this study, we examined the role of ADSC-derived exosomes on TXNIP inflammasome inhibition. To determine the effect of ADSC-exos on the pathological process of AAA, we injected ADSC-exos into the tail vein of AAA mice for 28 days. We found that TXNIP mRNA and protein levels in macrophages were markedly reduced in AAA mice tread with ADSC-exos. Also, we observed higher cumulative survival, lower elastin degradation, and reduced inflammatory cytokines in ADSC-exos mice compared to model mice. Further experimental data suggested that this process was regulated by exosomal miR-17-5p, which can suppress AAA progression by targeting the TXNIP-NLRP3 inflammasome. Interestingly, it has been demonstrated that macrophage-derived exosomes trigger MMP-2 expression in VSMC via JNK and p38 pathways, indicating another pathophysiological role of exosomes involved in the pathogenesis of AAA. In this present study, we found ADSC-exos containing different levels of miR-17-5p presented different therapeutic effects [35]. Specifically, the difference in the AAA incidence and between Exo^miR−17−5p inhibitor^ mice and Exo^miR−17−5p NC^ mice were observed, indicating exosomal miR-17-5p knockdown weakened the therapeutic efficacy of ADSC-exos. It indicated that the miR-17-5p-TXNIP axon played one of the essential roles in exosomes treatment for AAA. Taken together, these data provided the first functional evidence that miR-17-5p from ADSC-exos pivotally modulates TXNIP inflammasome activation in macrophages, suggesting exosomal miR-17-5p injection may become a future target for constraining the progression of AAA. What is noteworthy, miR-17-5p has been demonstrated to alleviates myocardial ischemia–reperfusion injury by regulating cell pyroptosis, which was a new paradigm of cell death for fighting against vascular diseases [42–44].

Cell pyroptosis is a caspase-1-dependent proinflammatory cell death characterized as cell swelling and dissolution of the cell membrane, which can be induced by triggering TXNIP-NLRP3 signaling [45]. This process is accompanied by the release of many inflammatory factors such as IL‐1β and IL‐18, which ultimately magnifies the local inflammatory response. Xiong et al. found that regulation of vascular smooth muscle cell pyroptosis could constrain AAA [46]. Moreover, macrophage pyroptosis has been suggested to be involved in AAA [47]. Our study elucidated that Ang II activated GSDMD in the macrophages of AAA mice, which was upregulated or downregulated by TXNIP artificial overexpression or knockdown in vitro. Moreover, the decline of GSDMD-N in the knockdown group was even more significant after ADSC-exos were added, while the upregulation of GSDMD was offset by ADSC-exos treatment. To sum up, our findings suggested that ADSC-exos might be a novel therapeutic approach for prevention of detrimental influences brought about by macrophages pyroptosis during AAA expansion (Fig. 8K).

Taken together, we inferred that miR-17-5p carried by ADSC-derived exosomes alleviated AAA progression by inhibiting the TXNIP-NLRP3 inflammasome signaling pathway. However, this study has some limitations. First, although the murine AAA model of Ang II infusion in ApoE^−/−^ C57BL/6 mice simultaneously receiving HFD for 28 days has been reported to mimic hypertension and hyperlipidemia of clinical AAA patients [48], there are other risk factors such as elastin degradation in AAA pathophysiological changes. Thereafter, further efforts should be made to improve the modeling method in order to better conform to the clinical situation of AAA. In addition, we artificially constructed activated or inactivated TXNIP at the cellular level to conduct a preliminary investigation of the underlying mechanisms in this project. Given the complex environment in vivo during the development of AAA, further TXNIP interference in AAA mice should be performed to confirm our deduction. Studies on TXNIP interference in vivo were carried out by our research team, which may help verify the functions and mechanisms of ADSC-derived exosomes in restraining AAA expansion via the TXNIP-NLRP3 signaling pathway.

## Conclusion

This study demonstrated the significant role of miR-17-5p from ADSC-exos in constraining AAA progression, inflammatory cytokines release, and TXNIP-NLRP3 inflammasome in vivo. In vitro further revealed that miR-17-5p could reduce macrophage pyroptosis during inflammation induced by Ang II by targeting TXNIP. Hence, miR-17-5p-rich ADSC-exos may become a new strategy in the clinical practice for using ADSC-derived exosomes in AAA treatment.

## Data Availability

Please contact the corresponding author for data requests.

## Abbreviations

ADSC: Adipose-derived mesenchymal stem cell
Exo: Exosome
miR-17-5p: MicroRNA-17-5p
AAA: Abdominal aortic aneurysm
Ang II: Angiotensin II
RT-qPCR: Reverse transcription-quantitative polymerase chain reaction
TXNIP: Thioredoxin-interacting protein
NLRP3: TXNIP-NOD-like receptor thermal protein domain associated protein 3
GSDMD: Gasdermin D
ELISA: Enzyme-linked immunosorbent assay
EVAR: Endovascular aneurysm repair
NTA: Nanoparticle tracking analysis
TEM: Transmission electron microscopy
DiR: 1,1’-Dioctadecyltetramethyl indotricarbocyanine iodide
TBST: Tris-buffered saline tween
ECL: Enhanced chemiluminescence
GAPDH: Glyceraldehyde‐3phosphate dehydrogenase
ANOVA: Analysis of variance
WT: Wild type
MUT: Mutated
NC: Negative control
Veh: Vehicle
